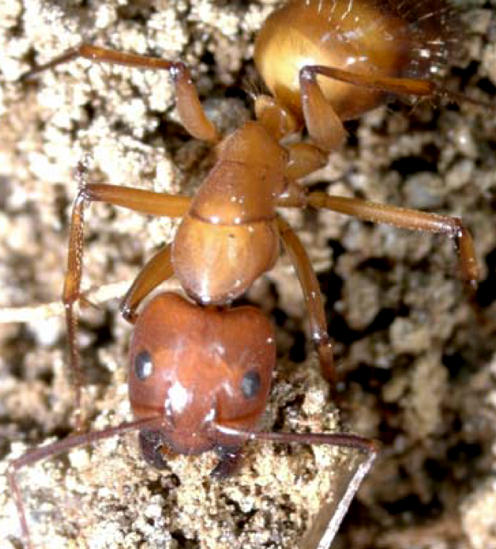# Correction

**DOI:** 10.1371/journal.pbio.0020201

**Published:** 2004-05-11

**In *PLoS Biology,* volume 2, issue 3:**

Table of Contents

Page iii

This photograph was used on the March 2004 Table of Contents, where Adam Lazarus, who generously supplied the image, should have been acknowledged. We apologize for this omission.[Fig pbio-0020201-g001]

**Figure pbio-0020201-g001:**